# Supplementary material for: Metagenomic and Metabolomic Analyses Reveal the Role of a Bacteriocin-Producing Strain of Enterococcus faecalis DH9003 in Regulating Gut Microbiota in Mice
Source: Microorganisms. 2025 Feb 8;13(2):372. doi: 10.3390/microorganisms13020372 (PMC11858018; doi:10.3390/microorganisms13020372)
Supplement: Supplementary file 1 [file microorganisms-13-00372-s001.zip › Figure S1.pdf]

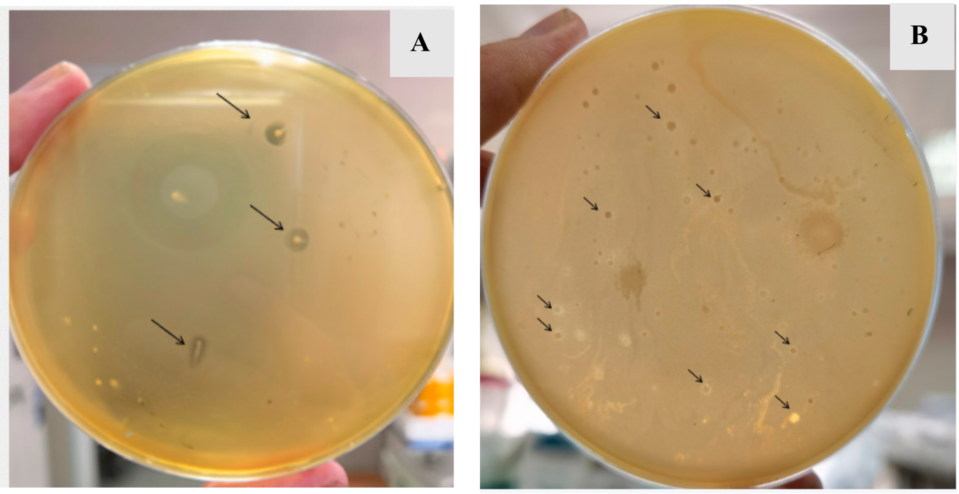

Figure S1 Bacterial strains isolated from faces samples showed antimicrobial activity against *Listeria monocytogenes* LFMT 2813. (A) faces samples diluted at 10 $^{-8}$, (B) faces samples diluted at 10 $^{-7}$.
