# Supplementary material for: Metagenomic and Metabolomic Analyses Reveal the Role of a Bacteriocin-Producing Strain of Enterococcus faecalis DH9003 in Regulating Gut Microbiota in Mice
Source: Microorganisms. 2025 Feb 8;13(2):372. doi: 10.3390/microorganisms13020372 (PMC11858018; doi:10.3390/microorganisms13020372)
Supplement: Supplementary file 1 [file microorganisms-13-00372-s001.zip › Table S1.pdf]

Table S1. Statistical analysis of sequencing quantity. HD presents as control group, HD_0, HD_3, HD_7, HD_14, HD_21, and HD_28 present samples collected at 0, 3, 7, 14, 21, and 28 days. YD presents as experimental group that treated with *E. faecalis* DH9003. YD_0, YD_3, YD_7, YD_14, YD_21, and YD_28 present samples collected at 0, 3, 7, 14, 21, and 28 days, respectively.

Sample IDs	Input	Filtered	Denoised	Merged	Non-chimeric
HD_0	94192	80471	78647	69855	43303
HD_3	107215	90584	88693	79798	45982
HD_7	112823	93801	91715	80413	48392
HD_14	102398	87900	85781	76914	53689
HD_21	94560	81550	79577	71278	47198
HD_28	92920	79193	77405	69517	47906
YD_0	101959	86502	84509	75541	51134
YD_3	108101	92464	90537	81712	51301
YD_7	108443	92960	91210	83590	47887
YD_14	102540	88070	86411	78989	56408
YD_21	91570	76447	75239	69309	46330
YD_28	103785	88811	87115	79328	55697
