# Supplementary material for: Metagenomic and Metabolomic Analyses Reveal the Role of a Bacteriocin-Producing Strain of Enterococcus faecalis DH9003 in Regulating Gut Microbiota in Mice
Source: Microorganisms. 2025 Feb 8;13(2):372. doi: 10.3390/microorganisms13020372 (PMC11858018; doi:10.3390/microorganisms13020372)
Supplement: Supplementary file 1 [file microorganisms-13-00372-s001.zip › Table S2.pdf]

Table S2. Statistics of metabolites identified by positive and negative ion modes.

Detection modes	Number of metabolites identified
Positive mode	1286
Negative mode	1140
