# Supplementary figures and images for: Metagenomic and Metabolomic Analyses Reveal the Role of a Bacteriocin-Producing Strain of Enterococcus faecalis DH9003 in Regulating Gut Microbiota in Mice
Source: Microorganisms. 2025 Feb 8;13(2):372. doi: 10.3390/microorganisms13020372 (PMC11858018; doi:10.3390/microorganisms13020372)

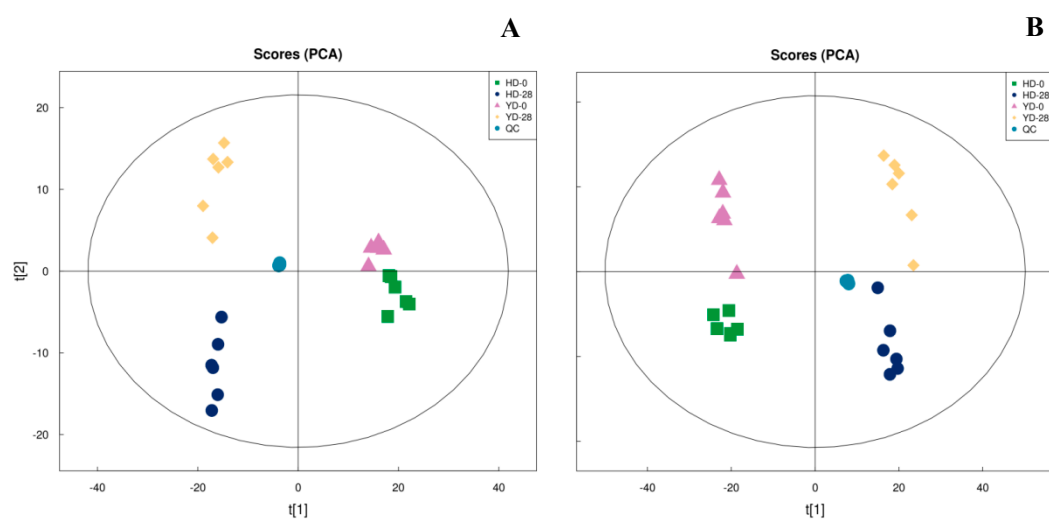

Figure S2. PCA analysis under total sample conditions, (A) positive ion mode, (B) negative ion mode.

Supplement: Supplementary file 1 [file microorganisms-13-00372-s001.zip › Figure S2.pdf]

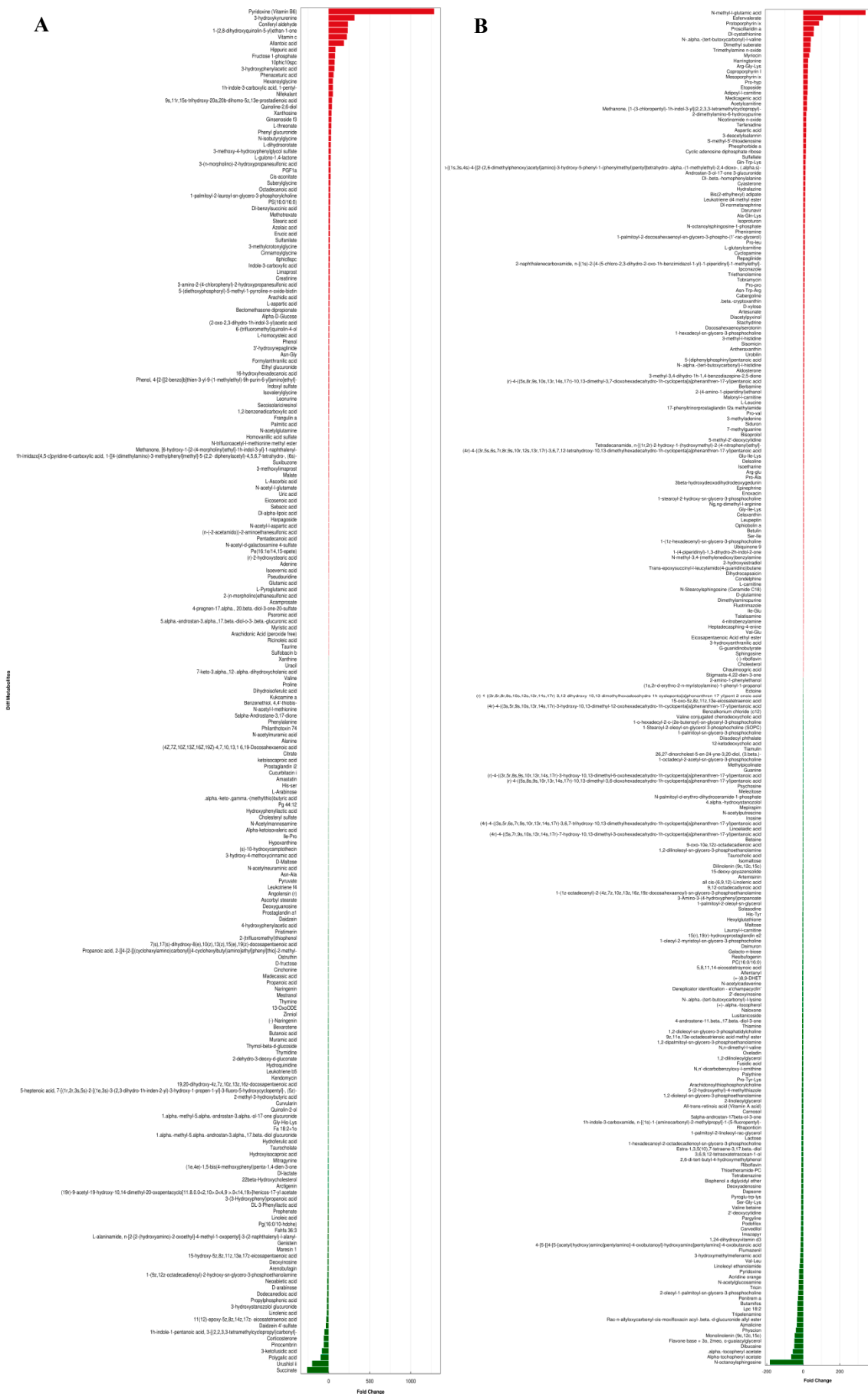

following treatment with *E. faecalis* DH9003. (A) negative ion mode, (B) positive ion mode.

Supplement: Supplementary file 1 [file microorganisms-13-00372-s001.zip › Figure S3.pdf]
